# Magnetic Carbon Porous Polymer Prepared from a New Suspended Emulsion for the Absorption of Heavy Metal Ions

**DOI:** 10.3390/polym17030257

**Published:** 2025-01-21

**Authors:** Shoulian Wei, Shenwei Huang, Jun Zhou, Chun Xiao, Jiangfei Cao, Jibo Xiao, Chunsheng Xie

**Affiliations:** 1 Medical School, Guangdong ATV College of Performing Arts, Zhaoqing 526631, China; weishlmary@126.com (S.W.); 13672876661@163.com (J.Z.); 2 College of Environmental and Chemical Engineering, Zhaoqing University, Zhaoqing 526061, China; 13711605527@163.com (S.H.); 13554301718@163.com (C.X.); jiangfei1989@yeah.net (J.C.); 3 Guangdong Provincial Key Laboratory of Eco-Environmental Studies and Low-Carbon Agriculture in Peri-Urban Areas, Zhaoqing University, Zhaoqing 526061, China; 4 College of Life and Environmental Science, Wenzhou University, Wenzhou 325035, China; jbxiao@wzu.edu.cn

**Keywords:** magnetic carbon polymer, heavy metals, adsorbent, wastewater

## Abstract

In this study, magnetic carbon nanopolymers (Fe_3_O_4_/C@PM) were synthesized by suspension polymerization using magnetic carbon nanoparticles as the matrix, 2-thiophene formaldehyde and acrylamide as the monomers, and ethylene glycol dimethacrylate (EGDMA) as the crosslinking agent. The obtained material was characterized using multiple techniques, including scanning electron microscopy (SEM), infrared spectroscopy (FTIR), X-ray diffraction (XRD), N_2_ adsorption–desorption, and thermogravimetric analysis (TGA). The adsorption effects of Zn^2+^, Cd^2+^, and Pb^2+^ in the mixed solution were evaluated using magnetic carbon nanoparticles (Fe_3_O_4_/C) and Fe_3_O_4_/C@PM as adsorbents. The adsorption isotherms, kinetic models, and cyclic regeneration of various metal ions, including Zn^2+^, Cd^2+^ and Pb^2+^, were studied. The results showed that the Fe_3_O_4_/C@PM maintained a slightly aggregated spherical morphology similar to Fe_3_O_4_/C and exhibited excellent adsorption capacity for all of Zn^2+^, Cd^2+^, and Pb^2+^, with maximum adsorption capacities of 343.3, 250.7, and 177.6 mg·g^−1^, respectively. The adsorption mechanisms were mainly based on the chemical interactions between metal ions and functional groups on the surface of polymers. The kinetic study revealed that the adsorption process followed a pseudo-second-order kinetic model. When Fe_3_O_4_/C@PM was reused five times, its adsorption rates for Zn^2+^, Cd^2+^, and Pb^2+^ remained above 81%, indicating its great potential for the treatment of wastewater containing Zn^2+^, Cd^2+^, and Pb^2+^.

## 1. Introduction

The contamination of water by metals has emerged as a significant environmental concern worldwide, posing risks to both marine life and human well-being [1,2]. Research has indicated that metal ions are resistant to biodegradation and cannot be processed by living organisms, leading to their accumulation. This in turn is detrimental to their central nervous system, reproductive systems, and organs such as the cardiovascular system, kidneys, and liver. In extreme situations, these substances may result in disorders related to cognitive development [3], potentially causing disabilities that last a lifetime [4]. Consequently, exploring methods to eliminate heavy metals from water systems is crucial for mitigating their detrimental impacts on the environment and ecological balance. To tackle this issue, various methods have been developed by scientists to effectively treat wastewater for the removal of heavy metals. Relevant methods encompass processes such as chemical precipitation [5], electrochemical treatment [6], ion exchange [7], adsorption [8], membrane filtration [9], and phytoremediation [10]. All these approaches have demonstrated considerable efficacy in managing wastewater contaminated with heavy metals, while the adsorption technique is particularly favored due to its straightforward operational process, high removal efficiency, significant impact, recyclability, eco-friendliness, low expenses, and strong adaptability to biological systems, garnering considerable interest in both academic and industrial circles [11,12]. The utilized adsorbent materials include carbon-based materials, such as nanoporous activated carbon, carbon nanotubes, and graphene oxide [13,14], which have attracted great interest. These materials have been popular research targets for their role as effective adsorbents in the removal of heavy metals from aqueous solutions, primarily owing to their cost-effectiveness, highly porous configuration, extensive surface area, and active surface functionalities. Biochar has become highly popular in recent years within the context of treating water contaminated by heavy metals, primarily owing to its economic efficiency and effectiveness in metal ion retention [15]. However, while biochar has demonstrated a notable ability to remove heavy metal ions from contaminated waters, its adsorption performance is often not as high as that of other established biosorbents like activated carbon. As a result, research has focused on enhancing the properties and structures of biochar through various modifications to improve its effectiveness in environmental remediation and its overall usefulness [16]. On the other hand, the separation of these non-magnetic carbon nanomaterials from water is challenging, leading to high costs and low recyclability, which are factors that hinder the overall effectiveness of biochar compounds in treating wastewater contamination.

The integration of magnetic nanomaterials leverages the benefits of both magnetic separation techniques and nanotechnology advancements, which can result in remarkable reusability after magnetic separation processes. Additionally, these materials are appreciated for their relatively large surface area, compatibility with biological systems, inert chemical properties, minimal toxicity, and ease of dispersion, features that render them highly effective for treating wastewater [17,18]. Researchers have engineered magnetic carbon nanomaterials along with their modified versions to facilitate the extraction of heavy metal ions from contaminated sources [19,20,21]. In Gabalda’s study, magnetic carbon nanomaterials were synthesized using electromagnetic induction heating and then utilized as nanoadsorbents for the extraction of Cr (VI) from water [22]. In a 25 mL aqueous solution with pH = 6 and 1 mg·L^−1^ Cr (VI), 25 mg of magnetic carbon nanomaterials were used as adsorbents for 30 min, and the efficiency of eliminating Cr (VI) exceeded 98%. In a study by Guo [23], Cd^2+^ was chosen as the template ion, and a magnetic ionic polymer (MIIP) consisting of graphitic carbon nitride (g-C_3_N_4_) was enhanced by incorporating polyn-isopropyl acrylamide through grafting. The results showed that in MIIP adsorbate aqueous solution with pH = 6 and an initial concentration of Cd^2+^ 250 mg·L^−1^, over a period of 180 min, the maximum adsorption capacity reached 184 mg·g^−1^. The mechanism of adsorption indicated that the initial specific uptake of Cd^2+^ by the ionic polymer is influenced by both its functional groups and the presence of ionic channels. Molecularly Imprinted Polymer has shown enhanced selectivity towards template ions, but its fabrication is a complex procedure, the elution of template ions proves challenging, the polymers may leak during the adsorption phase, and there remains a need to boost their adsorption capacity further. In order to address the above shortcomings, a series of polymeric molecularly imprinted polymers prepared from high internal phase emulsions (HIPEs) [24,25] or Pickering emulsions [26] were used as adsorbents to remove ions from aqueous solutions.

In this study, the synthesis of Fe_3_O_4_/C@PM, a type of magnetic carbon nanopolymer, achieved through a new suspension emulsion polymerization, was described. This process utilizes magnetic carbon nanoparticles as a foundational matrix, combined with 2-thiophenaldehyde and acrylamide serving as functional monomers, while ethylene glycol dimethacrylate (EGDMA) acts as the crosslinking agent. The fabrication of magnetic carbon nanopolymers, characterized by consistent particle size, effective dispersion, and stability, is achieved by utilizing Fe_3_O_4_/C nanoparticles as a stabilizing agent during the suspension emulsion polymerization process. The metal ion adsorption capability is enhanced by leveraging the strong binding properties of 2-thiophenaldehyde and acrylamide. A series of batch experiments are conducted to explore the influences of various factors, including pH, contact duration, starting ion concentration, and presence of competing ions, on the adsorption performance. To enhance the comprehension of the adsorption mechanisms, various parameters related to the adsorption behaviors of numerous metal ions, such as Zn^2+^, Cd^2+^, and Pb^2+^, are examined through isotherm analysis, kinetics models, and the assessment of recycling processes. The obtained data provide insights into the enhancement of future uses of innovative magnetic carbon nanopolymer materials.

## 2. Materials and Methods

### 2.1. Starting Materials

Hexahydrate of ferric chloride, heptahydrate of ferrous sulfate, sodium sulfite, disodium hydrogen phosphate, aqueous ammonia, sodium citrate in its dihydrate form, anhydrous ethanol, polyvinyl alcohol (PVA), sodium dodecyl sulfate, toluene, lead nitrate, nickel nitrate, zinc nitrate, and copper nitrate, all of analytical purity, were sourced from Guangzhou Chemical Reagent Factory. Cadmium nitrate, cobalt nitrate, 2-thiophenaldehyde, acrylamide, EGDMA, ammonium persulfate, and potassium persulfate were all of analytical grade and sourced from Aladdin Shanghai Co., LTD. (Shanghai, China). Solutions of Zn^2+^, Cd^2+^, Cu^2+^, Pb^2+^, Co^2+^, and Ni^2+^ at a concentration of 1000 μg·mL^−1^ were obtained from the National Nonferrous Metals and Electronic Materials Analysis and Testing Center located in Beijing, China. This study utilized Mill-Q ultra-pure water along with deionized water for all procedures.

### 2.2. Instruments

The instruments used in this work included the following: SQP precision scale (Sardorius Scientific Instrument (Beijing) Co., Ltd., Beijing, China), KXN-6450 DC regulated power supply (Shenzhen Zhaoxin Electronic Instrument Equipment Co., Ltd., Shenzh, China), JB50-S electric mixer (Shanghai Spectrum Biotechnology Co., Ltd., Shanghai, China), DF-101S constant temperature magnetic stirrer (Gongyi Yuhua Instrument Co., Ltd., Gongyi, China), DZF-6020 vacuum drying chamber (Shanghai Yiheng Scientific Instrument Co., Ltd., Shanghai, China), WE-2 constant temperature oscillator water bath (Tianjin Onuo Instrument Co., Ltd., Tianjin, China).

The material compositions were analyzed using an FTIR-8400S Fourier transformer infrared spectrometer. Differential thermogravimetric analysis was conducted using a DTG-60H (Shimadzu, Kyoto, Japan). The D8 Advance X-ray diffractometer (Bruker, Karlsruhe, Germany) was utilized for characterizing and analyzing the phases present in the materials. The materials’ surface characteristics were examined using a SUPRA 55 SAPPHIRE field emission scanning electron microscope (Carl Zeiss, Oberkochen, Germany). The materials were evaluated for their specific surface characteristics and pore configuration using a Brunauer–Emmett–Teller (BET) specific surface area and pore size analyzer (Autosorb-iQ, Quantachrome, Boynton Beach, FL, USA). Graphite furnace atomic absorption (GF-AAS, AA-7000, Shimadzu, Kyoto, Japan) was implemented to determine metal ion concentrations.

### 2.3. Synthesis of the Magnetic Carbon Nanopolymer

#### 2.3.1. Formulation of Carbon Nanosuspensions

The preparation of magnetic carbon nanosolutions was performed according to the reference [27]. Two carbon rods with a diameter of 1 cm were washed ultrasonically in distilled water and inserted into 40 mL PBS buffer with a concentration of 6 × 10^−5^ mol·L^−1^ and pH = 7. After connecting the DC regulated power supply, a method involving a constant current was utilized for ultrasonic electrolysis, with an electrode gap of 5 mm, current of 0.25 A, and ultrasonic power of 80 W, and the duration of electrolysis was set to 3 h. This resulted in a brown carbon nanosolution that was evenly distributed.

#### 2.3.2. Synthesis of Magnetizable Carbon Nanoparticles

Amounts of 0.3503 g FeCl_3_·6H_2_O, 0.1853 g FeSO_4_·7H_2_O, and 0.1388 g sodium citrate dihydrate were added into 40 mL carbon nanosolution and dissolved by ultrasound for 20 min to ensure they dispersed evenly. The mixture was placed in a 70 °C water bath, with the pH adjusted to 11 with ammonia water, stirred for 3 h, cooled, and magnetically separated. The products were cleaned with deionized water and anhydrous ethanol to a pH of 7, followed by vacuum drying at 60 °C for 12 h to obtain magnetic carbon nanoparticles (Fe_3_O_4_/C).

#### 2.3.3. Fabrication of Carbon-Based Magnetic Polymer Nanocomposites

Phase A for the aqueous solution involved introducing 1 g of PVA into a 500 mL round-bottom flask containing 200 mL of ultra-pure water. This mixture was heated to 90 °C while being stirred mechanically at 500 rpm. Once the PVA had dissolved entirely, 20 mL of 20% sodium dodecyl sulfate solution was added along with 2 g of sodium sulfite. After 30 min of stirring, the mixture was allowed to cool to 45 °C.

In a suitable 50 mL vessel, a mixture was prepared by gradually adding 2.5 mL of toluene, 1.2 mL of 2-thiophenaldehyde, 0.57 g of acrylamide, 4.0 mL of EGDMA, and 0.04 g of ammonium persulfate. The vessel was then subjected to ultrasonic treatment at 60 °C for a duration of 20 min to ensure full solubilization of the components.

**Figure 1:**
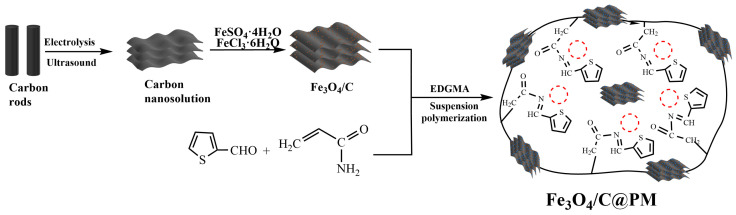
Schematic diagram of the preparation of magnetic carbon nanopolymer materials. (The red dashed circles mean the specific cavities for absorption.)

The synthesis of the magnetic carbon nanopolymer was achieved by the suspension polymerization technique with a water-to-oil ratio of 1:40. Specifically, 1.00 g Fe_3_O_4_/C and 0.04 g potassium persulfate were added to the aqueous phase A at 45 °C, to which approximately 5.4 mL of oil phase B was added progressively while maintaining mechanical stirring at a rate of 500 rpm for an hour to ensure the formation of a stable emulsion. Next, the polymerization process took place at 85 °C for 16 h to obtain a black solid product. The magnetic carbon nanopolymer (Fe_3_O_4_/C@PM) was then isolated after fine crushing and thorough rinsing in hot water for 4–5 cycles. To eliminate the remaining emulsion, Soxhlet extraction was performed using ethanol and an ethanol–acetic acid mixture for a duration of 24 h each. The magnetic carbon nanopolymer (Fe_3_O_4_/C@PM) was obtained by vacuum drying at 60 °C for 12 h. The aforementioned method of synthesizing magnetic carbon nanopolymer composites is displayed in Figure 1.

### 2.4. Adsorption Experiment

The efficacy of Fe_3_O_4_/C and Fe_3_O_4_/C@PM adsorbents in the removal of mixed metal ions from an aqueous solution was explored through competitive adsorption trials. A 10 mL standard solution containing a mixture of Zn^2+^ ions, Cd^2+^, Cu^2+^, Pb^2+^, Co^2+^, and Ni^2+^ in a concentration of 500 mg·L^−1^ and pH = 4, and 30 mg of either Fe_3_O_4_/C or Fe_3_O_4_/C@PM was added into a 100 mL conical flask, which was then placed in a temperature-controlled oscillator at 25 °C at 180 revolutions per minute for oscillatory adsorption for 1 h. After magnetic separation, the supernatant containing metal ions was subjected to analysis using flame atomic absorption spectrometry. The removal efficiency *R* (%) of each ion was determined by the following equation:

(1) $$R=\frac{c_{0}-c_{t}}{c_{0}}\times 100\%$$

where *c*_0_ and *c_t_* (measured in mg·mL^−1^) denote the initial metal ion concentration within the solution and its concentration post-adsorption after one hour, respectively; *V* (in mL) indicates the solution’s volume; m (in g) refers to the mass of the adsorbent used.

Batch experiments were conducted to investigate the ability of the adsorbents Fe_3_O_4_/C or Fe_3_O_4_/C@PM to adsorb metal ions, namely Zn^2+^, Cd^2+^, and Pb^2+^, while varying different conditions, such as the pH of the solution, the initial concentration of the metal ions, and the duration of adsorption.

The influence of the pH level of the solution was investigated by the following experiment: First, 30 mg of adsorbent was introduced into 10 mL of a solution that varied in pH from 2 to 7 adjusted with different concentrations of HNO_3_ and had a concentration of 500 mg·L^−1^ of Zn^2+^, Cd^2+^, and Pb^2+^. The mixture was then subjected to an adsorption reaction at 25 °C while being stirred at 100 rpm for one hour, followed by magnetic separation.

Static adsorption experiment: First, 15 mg adsorbent was dispersed in 10 mL solution with pH = 6.0 and containing Zn^2+^, Cd^2+^, and Pb^2+^ in the concentration range of 50~1000 mg·L^−1^. The adsorbent underwent reciprocating oscillation for 1 h at 25 °C and 100 rpm. The resulting adsorbent was collected by a magnet. The supernatant was analyzed by GF-AAS.

Dynamic adsorption experiment: First, 15 mg adsorbent was dispersed in 10 mL solution with pH = 6.0 and 400 mg·L^−1^ Zn^2+^, Cd^2+^, and Pb^2+^ at 25 °C, which was subjected to 0~60 min of oscillating adsorption at 100 rpm. The metal ion content in the solution was analyzed at different time points, allowing the adsorption quantity (*Q*) to be calculated at different times, and the adsorption kinetics curve was then drawn. The adsorption capacity *Q* (mg·g^−1^) was calculated by the following formula:

(2) $$Q=\frac{(c_{0}-c_{t})V}{m}$$

where *c*_0_ and *c_t_* (in mg·mL^−1^) denote the original concentration of metal ions present in the solution and the concentration recorded after t minutes of adsorption, respectively; *V* (in mL) signifies the volume of the solution; *m* (in grams) indicates the mass of the adsorbent used.

### 2.5. Regeneration Analysis

The reusable performance of the Fe_3_O_4_/C@PM adsorbent prepared with 0.1 mol·L^−1^ ethylenediaminetetraacetic acid (EDTA) as eluent was studied. For this experiment, 15 mg of the adsorbent was dispersed in 10 mL solution with pH = 6 and 400 mg·L^−1^ concentration of Zn^2+^. The adsorbent was treated with oscillations at a temperature of 25 °C and a frequency of 100 rpm for a duration of 30 min to achieve adsorption equilibrium. Subsequently, the levels of Zn^2+^ in this process were analyzed. The levels of Cd^2+^ and Pb^2+^ in the solution were determined using flame atomic absorption spectrometry. After the adsorbent became saturated with Zn^2+^, deionized water was used to rinse Cd^2+^ and Pb^2+^. After the addition of 30 mL of 0.1 mol·L^−1^ EDTA, the desorption of eluents occurred at a temperature of 45 °C while being agitated at 180 rpm for a duration of 20 min. This process was repeated with fresh eluents every 20 min until Zn^2+^ was no longer present. The presence of Cd^2+^ and Pb^2+^ was not observed. The regenerated material was subsequently rinsed with deionized water to prepare it for reuse in future applications. To assess the effectiveness of the synthesized adsorbent, the same adsorbent underwent an adsorption–desorption procedure six times.

## 3. Results and Discussion

### 3.1. Characterization of Fe_3_O_4_/C and Fe_3_O_4_/C@PM

**Figure 2:**
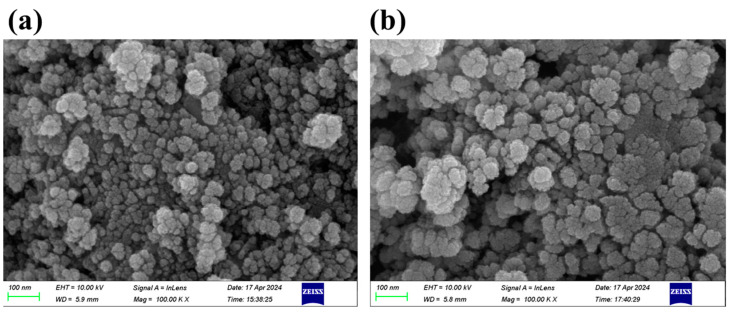
SEM images of Fe_3_O_4_/C (**a**) and Fe_3_O_4_/C@PM (**b**).

Figure 2 displays the scanning electron microscopy (SEM) images pertaining to Fe_3_O_4_/C and Fe_3_O_4_/C@PM. Figure 2a (Fe_3_O_4_/C) illustrates that the carbon nanoparticles produced through electrolysis exhibit an irregular lamellar structure, featuring pores that extend between the layers of carbon and housing spherical Fe_3_O_4_ particles within these carbon nanoparticles. Figure 2b (Fe_3_O_4_/C@PM) reveals that the polymer-coated Fe_3_O_4_/C has a dispersive arrangement of uneven layered granular structures characterized by a textured surface, promoting effective mass transfer.

**Figure 3:**
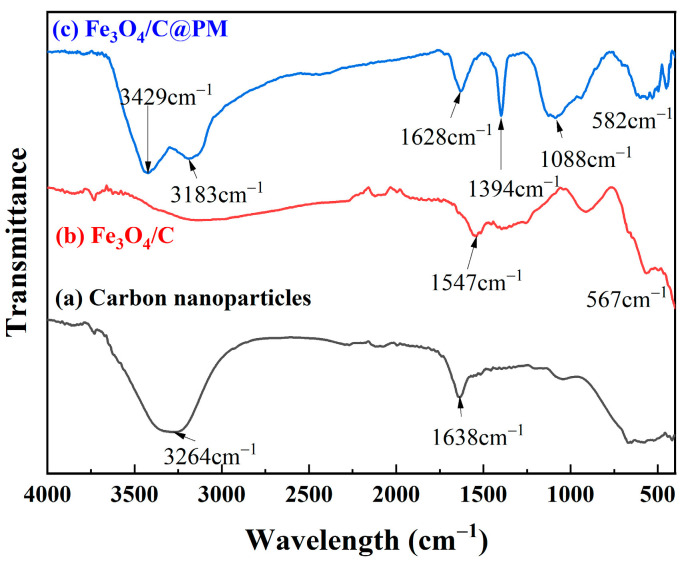
Infrared spectra of carbon nanoparticles (a), Fe_3_O_4_/C (b), and Fe_3_O_4_/C@PM (c).

The spectra for Fe_3_O_4_/C and Fe_3_O_4_/C@PM are illustrated in Figure 3. In Figure 3a, the spectrum corresponding to carbon nanoparticles synthesized through electrolysis is presented. The notable wide peaks observed in the range of 3400~2900 cm^−1^ correspond to the indicative stretching vibrations associated with -OH and -COOH groups, and 1638 cm^−1^ is the stretching vibration peak of C=O in the carboxyl group, which suggests that the carbon nanoparticles synthesized through electrolysis possess an abundance of -OH and -COOH functional groups [27]. The spectra in Figure 3b,c reveal a distinct Fe-O absorption band located between 567 and 580 cm^−1^, suggesting that ferric oxide is integrated within the carbon nanoparticles and polymers. The spectrum presented in Figure 3c is that of the Fe_3_O_4_/C@PM sample, where the broad peak observed at 1088 cm^−1^ corresponds to the stretching vibration associated with C-O-C, CS, and OH bonds. The characteristic absorption band at 1394 cm^−1^ is associated with CH_2_, while the broad and intense peak around 1628 cm^−1^ is linked to the -NH_2_, -C=C, -C=N, and -C=O stretching vibrations. The absorption signal observed between 2350 and 2600 cm^−1^ is associated with the stretching vibration of the sulfonyl group, and the stretching vibration peak at 3183 cm^−1^ is the stretching vibration peak of -CH=C on the thiophene ring. The strong absorption peaks in the range of 3300~3550 cm^−1^ correspond to the stretching vibration peaks of O-H and -NH_2_. The above results confirm the cross-linking reaction of thiophenic formaldehyde and acrylamide with EGDMA. It can be seen from the FTIR results that Fe_3_O_4_/C@PM has been successfully prepared.

**Figure 4:**
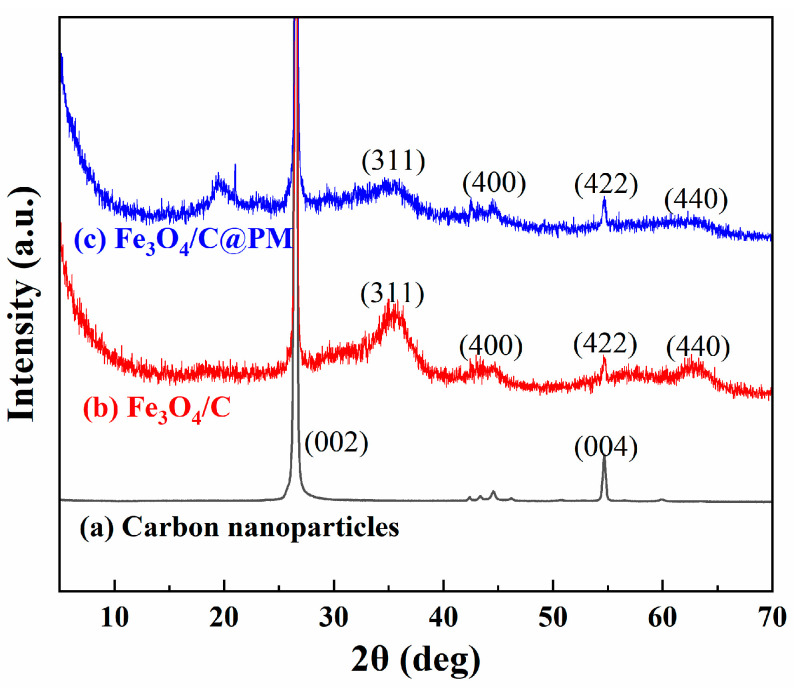
XRD patterns of carbon nanoparticles (a), Fe_3_O_4_/C (b), and Fe_3_O_4_/C@PM (c).

The XRD patterns of Fe_3_O_4_/C and Fe_3_O_4_/C@PM are presented in Figure 4, and the prepared carbon nanoparticles are shown in Figure 4a. The two sharp peaks at 2θ = 26.603° and 54.69° correspond to the (002) and (004) crystal plane diffraction peaks (JCPDS 75-1621) of graphitic carbon, respectively [28], suggesting that the structure of the prepared carbon nanoparticles resembles that of graphite. Regarding the graphs illustrated in Figure 4b,c, when compared to the reference diffraction pattern JCPDS No.19-062 for Fe_3_O_4_, diffraction peaks are observed at 2θ = 35.60°, 43.17°, 54.61°, and 62.65°, corresponding to the (311), (400), (422), and (440) planes of Fe_3_O_4_, respectively [23], suggesting that the synthesized composite materials incorporate Fe_3_O_4_ nanoparticles. In Figure 4c, a wide diffraction peak at 2θ = 19.43° is present, which aligns with the observed characteristic peak of the non-crystalline polymer mentioned in the reference [29], signifying the successful synthesis of Fe_3_O_4_/C@PM. The diminished intensity of the diffraction peaks associated with Fe_3_O_4_ in both Fe_3_O_4_/C and Fe_3_O_4_/C@PM can be explained by the presence of the carbon nanosheet and polymer coating [30]. These findings are in good agreement with the FTIR analysis results.

Utilizing TGA allows for the quantitative analysis of the proportions of carbon nanoparticles and polymers present in the composite material. In Figure S1, the TGA profiles of Fe_3_O_4_/C and Fe_3_O_4_/C@PM are illustrated. The TGA profile corresponding to Fe_3_O_4_/C (shown in Figure S1a) indicates a mass reduction of 9.65% when analyzed over the temperature interval of 25–240 °C, which results from the loss of both absorbed moisture and structural water present within the sample. At 240–700 °C, the loss of weight gradually accelerates as the temperature rises. This phenomenon can be largely attributed to the elimination of carboxyl and hydroxyl groups that are attached to the surface. Once the temperature exceeds 700 °C, a notable reduction in weight occurs due to the thermal oxidative breakdown of carbon nanoparticles. Within the temperature range of 240–800 °C, the reduction in mass observed for carbon nanoparticles is recorded at 12.93%. In comparison to Fe_3_O_4_/C, in the range of 25–240 °C, Fe_3_O_4_/C@PM experiences a weight reduction of 10.03% (Figure S1b). Within the range of 240–800 °C, a considerable mass reduction (41.43%) occurs owing to the breakdown of the polymer and the thermal degradation of carbon nanoparticles. These findings indicate that Fe_3_O_4_/C@PM exhibits strong thermal stability when subjected to a decomposition temperature of 240 °C.

The isotherms for nitrogen adsorption and desorption of Fe_3_O_4_/C and Fe_3_O_4_/C@PM are depicted in Figure S2. These are characteristic of type IV, displaying a distinct hysteresis loop at elevated pressures, suggesting a mesoporous architecture within the materials. The pore size distributions for Fe_3_O_4_/C and Fe_3_O_4_/C@PM are illustrated in Figure S3, where the primary pore dimensions for Fe_3_O_4_/C range from 0.3 nm to 5 nm, with a mean pore dimension of 0.622 nm. The primary pore size of Fe_3_O_4_/C@PM is noted to vary between 0.5 nm and 10 nm, with a significant concentration of mesoporous sizes observed around 3.5 nm. The average pore diameter is 0.823 nm, with mesopores predominantly found near 5 nm, suggesting the presence of numerous micropores and mesopores within the structure. According to the findings in Figure S3, the measured values of specific surface area for Fe_3_O_4_/C and Fe_3_O_4_/C@PM, obtained through multi-point BET, are 239.2 m^2^·g^−1^ and 348.5 m^2^·g^−1^, respectively, in line with total pore volumes recorded at 0.228 and 0.3017 cm^3^·g^−1^, respectively. It is evident from these data that Fe_3_O_4_/C@PM exhibits a greater specific surface area, while Fe_3_O_4_/C has a larger total pore volume and finer pore dimensions. This phenomenon can be attributed to the processes involved in suspension emulsion polymerization: the dispersion of Fe_3_O_4_/C becomes more uniform, resulting in an improved dispersion of Fe_3_O_4_/C@PM, which leads to a growth in the available surface area. The enlargement of pore dimensions and overall pore capacity is the result of voids created by tiny organic solvent particles and dispersed emulsified droplets.

### 3.2. Adsorption of Fe_3_O_4_/C@PM

#### 3.2.1. Influence of pH Value

**Figure 5:**
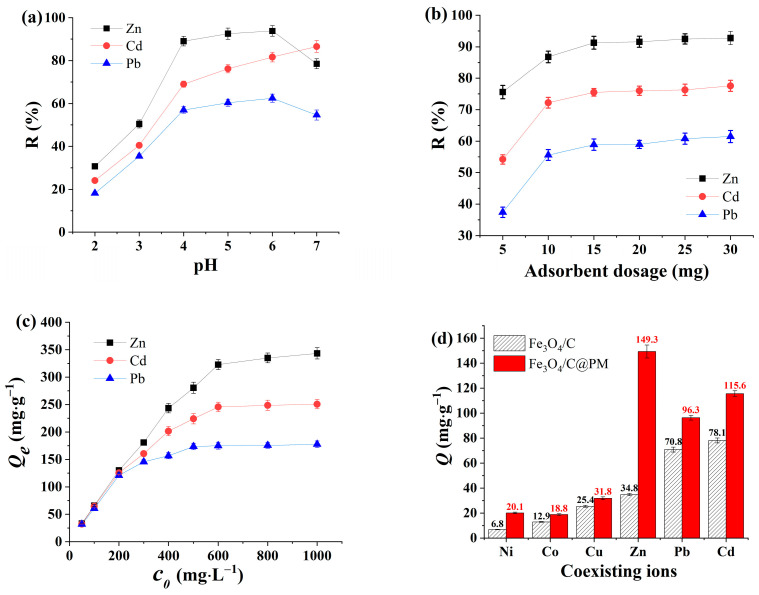
Effect of pH (**a**) and adsorbent dosage (**b**) on the removal of different metal ions and effect of initial concentration (**c**), and coexisting ions (**d**) on adsorption capacity.

The acidity level of the medium is crucial in the process of substance adsorption, as it influences not only the reactions occurring with metal ions within the medium but also the charge properties of the adsorbent material. The effectiveness of 30 mg Fe_3_O_4_/C@PM composite in removing 500 mg/L concentrations of Zn^2+^, Cd^2+^, and Pb^2+^ was evaluated within a pH range of 2 to 7 in a 10 mL solution. As shown in Figure 5a, the highest removal efficiencies for Zn^2+^, Cd^2+^, and Pb^2+^ are observed at pH levels of 6, 7, and 6, respectively. Between pH levels 2 and 4, a notable increase in the Zn^2+^, Cd^2+^, and Pb^2+^ removal rate occurs with the rising pH. Between pH levels of 4 and 6, the rate of removal for each ion continues to rise gradually, ultimately stabilizing. As the pH of the solution increases at lower pH levels (pH < 6), the amino and thiophene functional groups on the Fe_3_O_4_/C@PM surface become highly protonated, which inhibits the entry of Zn^2+^, Cd^2+^, and Pb^2+^ ions into the adsorbent’s surface. At the same time, a reduction occurs in the protonation of the functional groups present on the Fe_3_O_4_/C@PM surface, leading to a decrease in electrostatic repulsion of adsorbent’s surface with Zn^2+^, Cd^2+^, and Pb^2+^, which subsequently reduces the removal efficiency. When the pH exceeds 6, the formation of insoluble compounds such as Zn (OH)_2_ and Pb (OH)_2_ occurs in the solution, which leads to a decrease in the levels of Zn^2+^ and Pb^2+^ attached to the adsorbent, ultimately resulting in a notable drop in the removal efficiency for these ions. According to the above observations, a solution pH of 6 was selected for the subsequent experiments.

#### 3.2.2. Impact of Varying Concentrations of Fe_3_O_4_/C@PM on Removal Efficacy

The efficacy of Fe_3_O_4_/C@PM, in amounts ranging from 5 to 30 mg, in eliminating Zn^2+^, Cd^2+^, and Pb^2+^, separately, in 10 mL 500 mg·L^−1^ solutions was assessed. The pH was set to 6 for reasons discussed above, as illustrated in Figure 5b. With increasing amounts of Fe_3_O_4_/C@PM added, there was a substantial rise in removal rates of Zn^2+^, Cd^2+^, and Pb^2+^. Nevertheless, once the amount of Fe_3_O_4_/C@PM surpassed 10 mg, the increase in the ion removal efficacy noticeably diminished, which could be attributed to the merging or clustering of adsorption sites with the increasing quantity of adsorbents, leading to their reduced utilization efficiency. Beyond a dose of 15 mg adsorbent, the increase in the ion removal efficiency remained minimal, nearing its optimal level, suggesting that the adsorption had achieved a state of saturation equilibrium. Consequently, the ideal concentration of Fe_3_O_4_/C@PM was determined as 1.5 g·L^−1^.

#### 3.2.3. Effect of Initial Concentration of Heavy Metal Pollutants

In order to assess the capability of Fe_3_O_4_/C@PM in adsorbing Zn^2+^, Cd^2+^, and Pb^2+^, the influence of varying initial concentrations of Zn^2+^ on the adsorption process was investigated, and the influence of both Cd^2+^ and Pb^2+^ present on the adsorption ability of Zn^2+^ was examined at a temperature of 25 °C and a pH level of 6.0. The results are illustrated in Figure 5c, spanning an initial concentration spectrum of 50 to 200 mg·L^−1^ Zn^2+^. It can be seen that as the initial levels of Zn^2+^ increase, the capacity for its adsorption by Fe_3_O_4_/C@PM rises, while the removal of Cd^2+^ and Pb^2+^ shows a linear increase until the removal rate of Pb^2+^ is nearly complete, at which point there is still and Zn^2+^ and Cd^2+^ in the solution. This observation is attributed to the ample active sites present on the surface of Fe_3_O_4_/C@PM, and that the insufficient amount of metal ions present fails to occupy all these active sites effectively. When the starting concentration of Zn^2+^ ranges from 200 to 500 mg·L^−1^, the maximum capacity for its adsorption, the adsorption levels of Cd^2+^ and Pb^2+^ display a gradual and slow increase due to the gradual reduction in the number of available active sites on Fe_3_O_4_/C@PM. Upon raising the initial concentration of Zn^2+^ to 600 mg·L^−1^, the adsorption process appears to reach a level of completion, with the capacity stabilizing at a consistent value. This shows that metal ions have fully occupied the active sites of Fe_3_O_4_/C@PM. The peak adsorption capacities for Zn^2+^, Cd^2+^, and Pb^2+^ in connection with Fe_3_O_4_/C@PM reach 343.3, 250.7, and 177.6 mg·g^−1^, respectively.

#### 3.2.4. Selectivity of Adsorption

Various types of heavy metal ions, such as Zn^2+^, Cd^2+^, Cu^2+^, Pb^2+^, Co^2+^, and Ni^2+^, frequently exist together in both natural and industrial effluents. The impacts of 30 mg Fe_3_O_4_/C and Fe_3_O_4_/C@PM on the simultaneous presence of these metal ions were evaluated in a volume of 10 mL, pH = 4.0, with concentrations set at 500 mg·L^−1^. As illustrated in Figure 5d, in comparison to Fe_3_O_4_/C, the Fe_3_O_4_/C@PM composite exhibits an enhanced ability to adsorb various metal ions present simultaneously, and it demonstrates significant effectiveness in eliminating them from contaminated water. This is particularly the case for the uptake and elimination of Zn^2+^ ions, and the removal of Cd^2+^ and Pb^2+^ can also be enhanced. This might result from the increased specific surface area of Fe_3_O_4_/C@PM, a greater number of active sites, along with a richer presence of surface functional thiophene and amide groups compared to Fe_3_O_4_/C; therefore, it displays enhanced effects of electrostatic interactions and coordination with Zn^2+^, Cd^2+^, and Pb^2+^. The adsorbent chosen for the optimization of adsorption conditions and the investigation of the adsorption mechanism concerning Zn^2+^, Cd^2+^, and Pb^2+^ in water was Fe_3_O_4_/C@PM.

#### 3.2.5. Analysis of Adsorption Isotherms

In order to enhance the assessment of the adsorption potential of Fe_3_O_4_/C@PM regarding Zn^2+^, Cd^2+^, and Pb^2+^, the experimental data on adsorption equilibrium were analyzed using the Langmuir and Freundlich isotherm models [31]. The corresponding formulas can be articulated as follows:

The equation representing the Langmuir model in linear form:

(3) $$\frac{c_{e}}{Q_{e}}=\frac{1}{Q_{m}K_{L}}+\frac{c_{e}}{Q_{m}}$$

The Freundlich model is represented by a linear equation:

(4) $$\ln Q_{e}=\ln K_{F}+\frac{1}{n}\ln c_{e}$$

In Equation (4), *c_e_* (mg·L^−1^) denotes the concentration of metal ions once the adsorption process has attained equilibrium; *Q_e_* and *Q_m_* (mg·g^−1^) signify the adsorption capacities at the equilibrium state and at the point of saturation, respectively; *K_L_* and *K_F_* (L·mg^−1^) correspond to the isothermal adsorption constants defined by the Langmuir and Freundlich models, respectively; the variable *n* functions as the Freundlich adsorption constant and is indicative of the intensity of the adsorption process.

**Figure 6:**
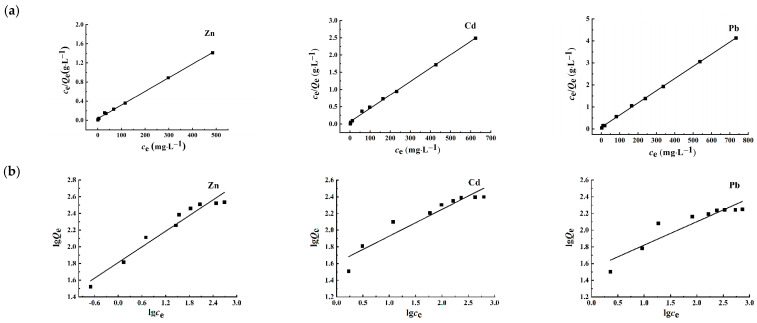
Langmuir isotherm diagram (**a**) and Freundlich isotherm diagram (**b**) of the adsorption of Zn^2+^, Cd^2+^, and Pb^2+^ by Fe_3_O_4_/C@PM.

Figure 6 illustrates the adsorption isotherms derived from the Langmuir and Freundlich models. The parameters associated with both models are presented in Table 1, where the correlation coefficient (R^2^) derived from the linear equation of the Langmuir model exceeds that of the Freundlich model. This suggests that the Langmuir isotherm provides a more accurate representation of the adsorption dynamics of heavy metals on Fe_3_O_4_/C@PM. It can be concluded that the nature of adsorption follows a monolayer pattern, which encompasses both physical and chemisorption processes. The highest adsorption levels (*Q_m_*) for Zn^2+^ and the calculated values for Cd^2+^ and Pb^2+^, derived from the Langmuir adsorption theory, amount to 349.65, 256.41, and 180.83 mg·g^−1^, respectively, which closely matches the experimental observation of 343.3, 250.7, and 177.6 mg·g^−1^. In addition, the Freundlich constant *n* falls within the range of 0 to 1, suggesting the existence of a significant attraction between the adsorbent and the metal ion, facilitating the adsorption process.

In order to assess the effectiveness of the Fe_3_O_4_/C@PM developed in this study in the adsorption of Zn^2+^, Cd^2+^, and Pb^2+^, a comparison of the maximum adsorption capabilities of previously studied magnetic carbon polymer adsorbents is provided in Table 2. The data indicate that the adsorbents developed in this research exhibit a notably higher maximum capacity for adsorbing Zn^2+^, Cd^2+^, and Pb^2+^ compared to certain other materials, showcasing their enhanced characteristics and significant potential for effective application in treating contaminated wastewater to eliminate these metal ions.

#### 3.2.6. Analysis of Adsorption Kinetics

In order to assess the effectiveness of metal ion capture by the adsorbent, the effect of exposure duration on the adsorption process was examined, and the findings are illustrated in Figure S4. As the duration of adsorption increases, the adsorption capacity increases swiftly, reaching a state of equilibrium after 20 min. Once this balance has been attained, the peak adsorption capacity *Q_e_* of Fe_3_O_4_/C@PM for Zn^2+^ ions, Cd^2+^, and Pb^2+^ are equal to 240, 204.6, and 139.6 mg·g^−1^, respectively. The contents of Zn^2+^, Cd^2+^, and Pb^2+^ are as high as 90%, 76.7%, and 52.3%, respectively, showing a notable capability for absorbing Zn^2+^ ions, attributed to the significant specific surface area and the abundance of functional groups on Fe_3_O_4_/C@PM, enhancing its capacity to capture Cd^2+^ and Pb^2+^, as well as offering numerous active sites and a robust ability for metal coordination.

In order to investigate how Zn^2+^, Cd^2+^, and Pb^2+^ are adsorbed by Fe_3_O_4_/C@PM, researchers can employ a quasi-first-order and a quasi-second-order kinetic model together to analyze the obtained kinetic data. The equations representing the linear forms of the two models are as follows:

Pseudo-first-order model:

(5) $$\ln(Q_{e}-Q_{t})=\ln Q_{e}-K_{1}t$$

Pseudo-second-order framework:

(6) $$\frac{t}{Q_{t}}=\frac{1}{K_{2}Q_{e}^{2}}+\frac{1}{Q_{e}}t$$

**Figure 7:**
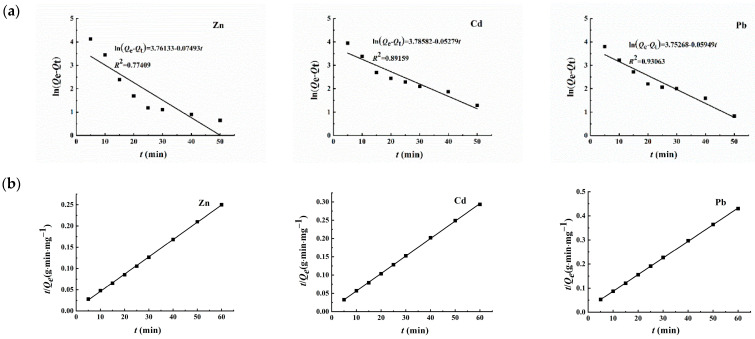
Dynamic fitting diagram of the quasi-first-order (**a**) and quasi-second-order (**b**) dynamic model.

In the above equations, *Q_e_* and *Q_t_* (mg·g^−1^) indicate the equilibrium adsorption capacity and the time (*t*, in minutes), respectively; the terms *K*_1_ (min^−1^) and *K*_2_ (mg·g^−1^·min^−1^) denote the rate constants for quasi-first-order and quasi-second-order adsorption processes, respectively; t signifies the duration of adsorption (in minutes). The model parameters and resulting graphs can be found in Table 3 and Figure 7, respectively.

### 3.3. Desorption and Reuse

**Figure 8:**
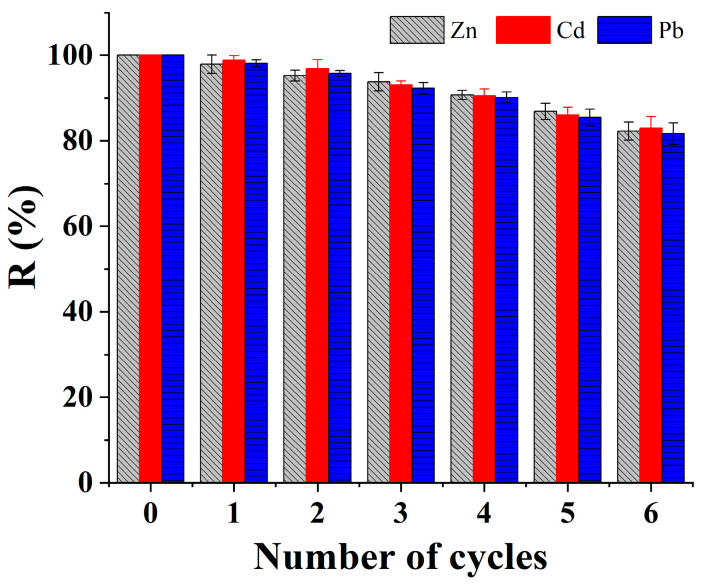
Adsorption rates on regenerated Fe_3_O_4_/C@PM for Zn^2+^, Cd^2+^, and Pb^2+^.

The reusability of adsorbents plays a crucial role in determining their suitability for practical applications. In this study, 0.1 mol·L^−1^ EDTA was utilized as the eluent. The reusability of the synthesized adsorbent Fe_3_O_4_/C@PM was assessed, and the findings are illustrated in Figure 8. The capability of Fe_3_O_4_/C@PM in adsorbing Zn^2+^ was evaluated. The concentrations of Cd^2+^ and Pb^2+^ showed minimal reduction despite increasing the number of cycles of adsorption and desorption. Following six cycles of this process, during which Zn^2+^ was completely adsorbed, the removal efficiency for Cd^2+^ and Pb^2+^ on Fe_3_O_4_/C@PM remained above 81%. The capacity for adsorption could achieve 196.4, 162.9 and 107.9 mg·g^−1^, respectively, for these three metal ions, showing favorable properties regarding both reusability and stability, especially in the context of Zn^2+^ adsorption. Thus, Fe_3_O_4_/C@PM demonstrates significant potential as an effective adsorbent for the repeated extraction of wastewater pollutants, particularly those containing Zn^2+^, Cd^2+^, and Pb^2+^.

## 4. Conclusions

In this study, the synthesis of polymeric materials with magnetic properties was accomplished using 2-thiophene formaldehyde alongside acrylamide as the primary monomer. Carbon nanoparticles with magnetic properties were utilized as a base material, EGDMA as a crosslinker, and the suspension polymerization technique was employed with a water-to-oil ratio of 1:40. The resulting magnetic carbon nanopolymer exhibited an irregular layered flower-like morphology characterized by a rough surface, good dispersibility, magnetic properties, and robust stability. This material boasts a specific surface area of 348.7 m^2^·g^−1^, with the pore diameter predominantly falling within the range of 0.5–10 nm. The highest absorption capacities of Fe_3_O_4_/C@PM for Zn^2+^, Cd^2+^, and Pb^2+^ metal ions were recorded at 343.3, 250.7, and 177.6 mg·g^−1^, respectively. For the uptake characteristics of Fe_3_O_4_/C@PM, the kinetics of Zn^2+^, Cd^2+^, and Pb^2+^ adsorption aligned with the quasi-second-order model, and the adsorption characteristics of heavy metal ions on Fe_3_O_4_/C@PM align with the Langmuir adsorption isotherm, suggesting that the adsorption mechanism is monolayer chemisorption. Overall, the Fe_3_O_4_/C@PM serves as a novel and highly effective material for adsorption, which holds significant promise for the remediation of wastewater contaminated with Zn^2+^, Cd^2+^, and Pb^2+^.

## Figures and Tables

**Table 1:** Thermodynamic model fitting parameters of Zn^2+^, Cd^2+^, and Pb^2+^ by Fe_3_O_4_/C@PM.

Metal Ion	Langmuir			Freundlich		
	*Q_m_/*(mg·g^−1^)	*K*_L_/(min^−1^)	*R* ^2^	1/*n*	*K*_F_/(mg·g^−1^·min^−1^)	*R* ^2^
Zn^2+^	349.65	0.0853	0.9980	0.31315	64.916	0.9488
Cd^2+^	256.41	0.0619	0.9972	0.32003	40.635	0.8922
Pb^2+^	180.83	0.0702	0.9994	0.28106	34.657	0.8401

**Table 2:** Comparison of properties of different magnetic carbon composite adsorbents.

Adsorbent	Preparation Method	*Q*_m_ (mg·g^−1^)	Reference
Magnetic graphitic carbon nitride	Multi-step and surface imprinting technology	258.35 for Cd^2+^	[23]
Fe_3_O_4_/BC/AC	Multi-step reaction	161.78 for Pb^2+^	[32]
Fe_3_O_4_@C nanoparticles modified with–SO_3_H and –COOH Groups	Multi-step reaction	90.7, 83.1 and 39.7 mg·g^−1^ for Pb^2+^, Hg^2+^ and Cd^2+^	[33]
mGO/CS mGO/PA	Multi-step reaction	110.84 and 118.44 mg·g^−1^ for Pb^2+^ were obtained for mGO/CS and mGO/PA	[34]
Carbon coating and polyacrylamide functionalization of Fe_3_O_4_ nanoparticles	Hydrothermal technique	231.7 mg·g^−1^ for Cd^2+^	[35]
3D Fe_3_O_4_@MWCNT-CdIIP	Multi-step reaction	109 mg·g^−1^ for Cd^2+^	[36]
Fe_3_O_4_/C@PM	Suspension polymerization	349.65, 256.41, 180.83 mg·g^−1^ for Zn^2+^, Cd^2+^, Pb^2+^	This work

**Table 3:** Fitting parameters of the quasi-first-order and quasi-second-order kinetic models.

Metal Ion	Quasi-First-Order			Quasi-Second-Order		
	*Q*_e_ (mg·g^−1^)	*K*_1_ (min^−1^)	*R* ^2^	*Q*_e_ (mg·g^−1^)	*K*_2_ (mg·g^−1^·min^−1^)	*R* ^2^
Zn^2+^	42.98	0.07493	0.77409	246.3	0.00291	0.99959
Cd^2+^	44.04	0.05279	0.89159	209.2	0.00260	0.99975
Pb^2+^	42.60	0.05949	0.93063	144.9	0.00258	0.99983

## Data Availability

The original contributions presented in this study are included in the article/supplementary material. Further inquiries can be directed to the corresponding author.

## Supplementary Materials

The following supporting information can be downloaded at: https://www.mdpi.com/article/10.3390/polym17030257/s1, Figure S1. Thermogravimetric analyses of Fe_3_O_4_/C (a) and Fe_3_O_4_/C@PM (b); Figure S2. Nitrogen adsorption-desorption isotherms for Fe_3_O_4_/C (a) and Fe_3_O_4_/C@PM (b); Figure S3. Aperture distributions of Fe_3_O_4_/C (a) and Fe_3_O_4_/C@PM (b); Figure S4. Adsorption kinetics curve.
