# Supplementary material for: Magnetic Carbon Porous Polymer Prepared from a New Suspended Emulsion for the Absorption of Heavy Metal Ions
Source: Polymers (Basel). 2025 Jan 21;17(3):257. doi: 10.3390/polym17030257 (PMC11820378; doi:10.3390/polym17030257)
Supplement: Supplementary file 1 [file polymers-17-00257-s001.zip › polymers-3318338-supplementary.pdf]

Supplementary Materials:

Figure S1. Thermogravimetric analyses of Fe_3O_4/C (a) and Fe_3O_4/C@PM (b).

Figure S2. Nitrogen adsorption-desorption isotherms for Fe_3O_4/C (a) and Fe_3O_4/C@PM (b).

Figure S3. Aperture distributions of Fe_3O_4/C (a) and Fe_3O_4/C@PM (b).

Figure S4. Adsorption kinetics curve.

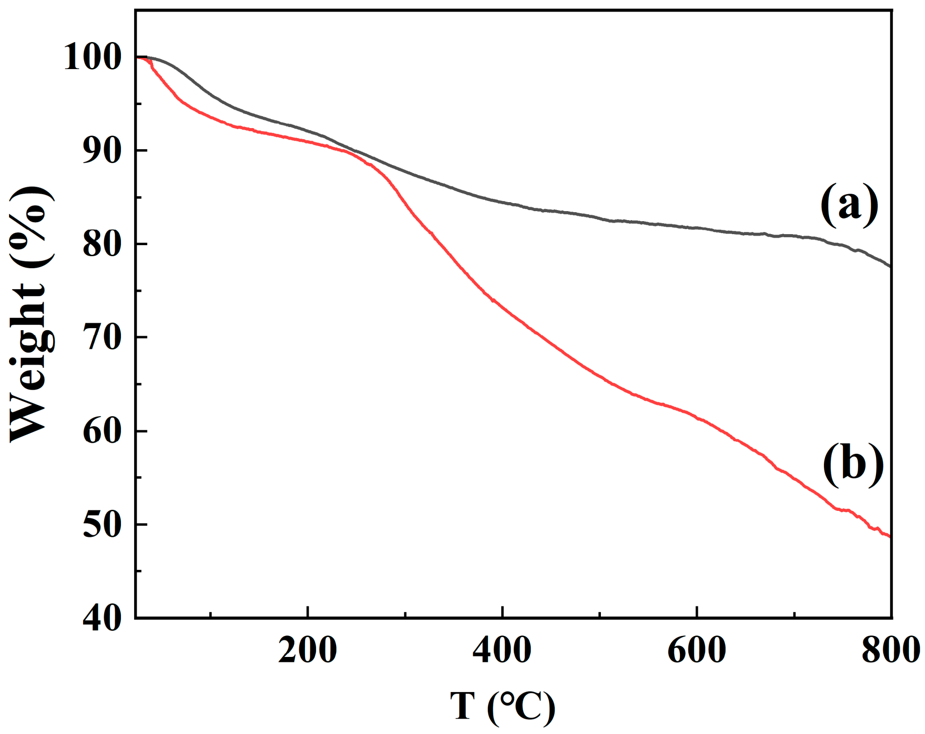

Figure S1. Thermogravimetric analyses of Fe_3O_4/C (a) and Fe_3O_4/C@PM (b).

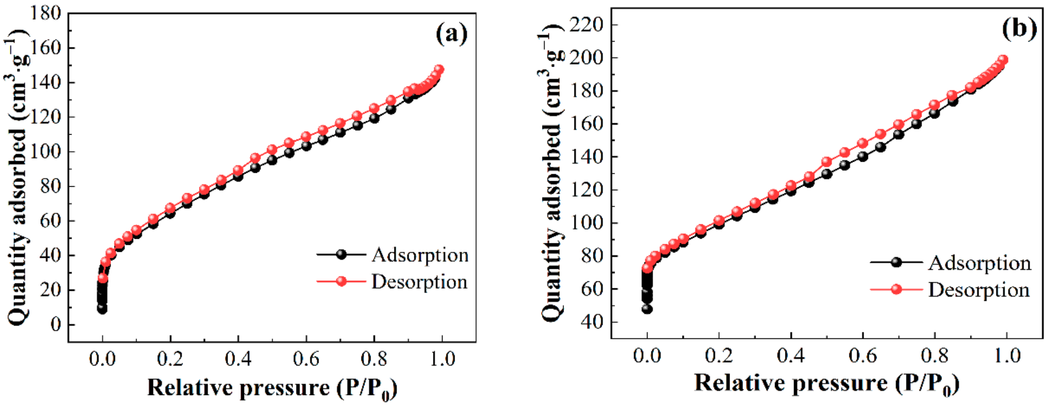

Figure S2. Nitrogen adsorption-desorption isotherms for Fe_3O_4/C (a) and Fe_3O_4/C@PM (b).

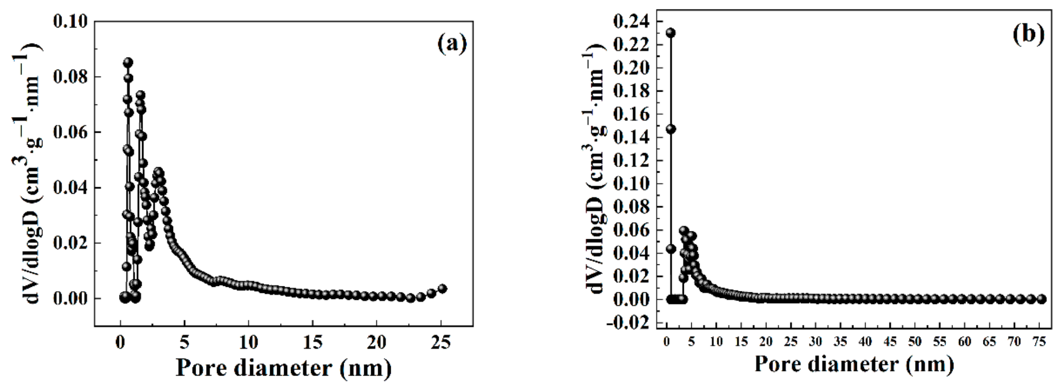

Figure S3. Aperture distributions of Fe_3O_4/C (a) and Fe_3O_4/C@PM (b).

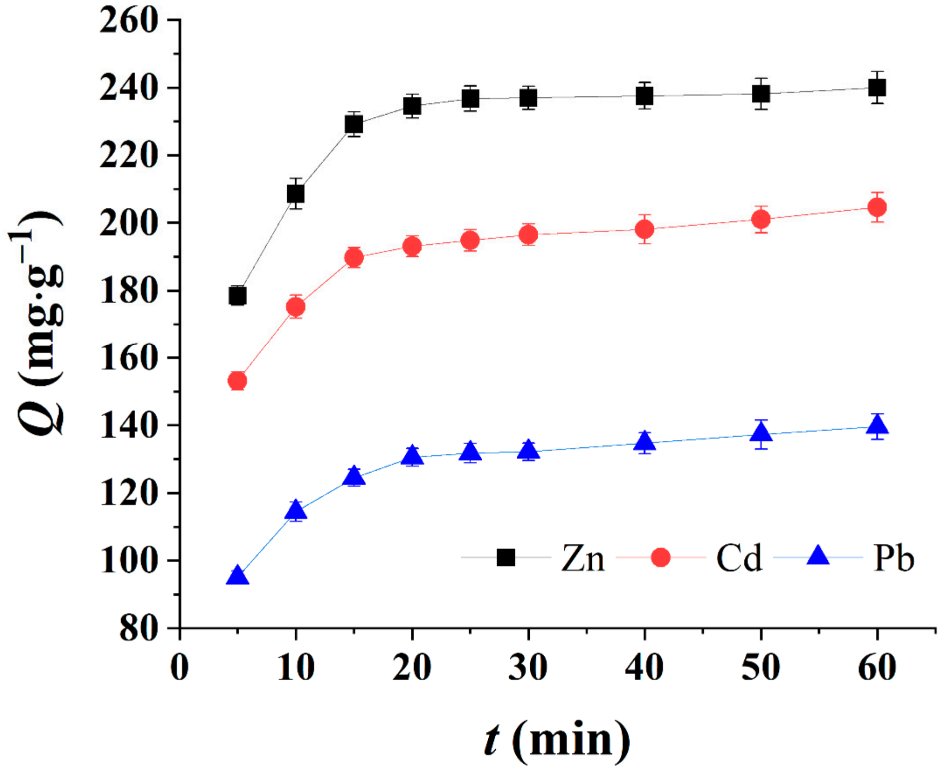

Figure S4. Adsorption kinetics curve.
